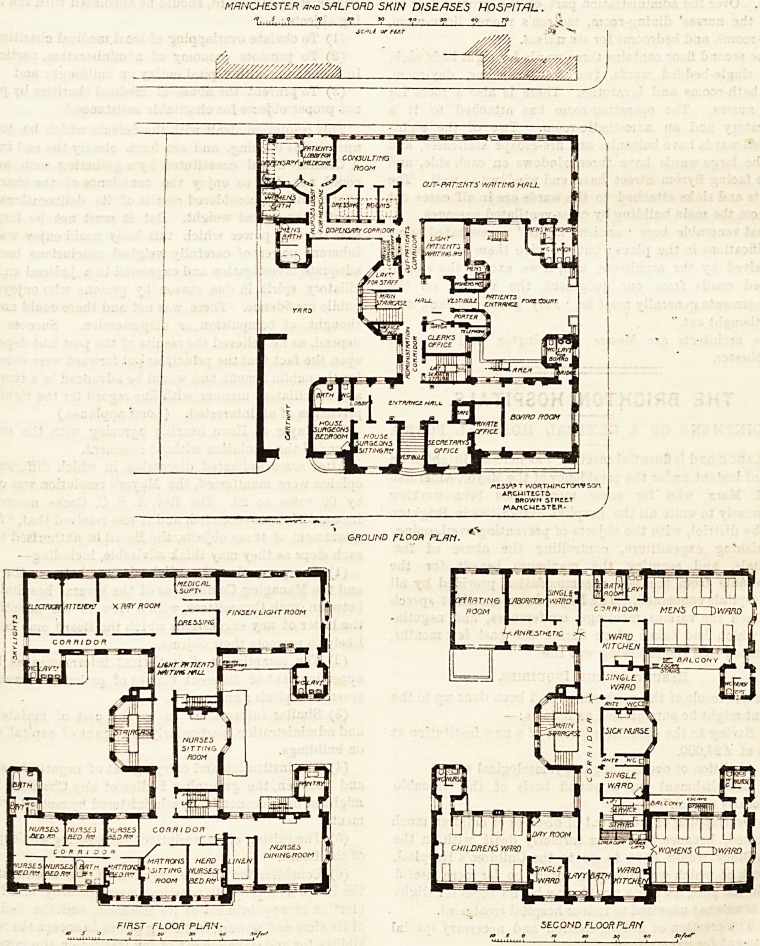# Manchester Hospital for Skin Diseases

**Published:** 1904-10-08

**Authors:** 


### HOSPITAL ADMINISTRATION.

### CONSTRUCTION AND ECONOMICS.

### MANCHESTER HOSPITAL FOR SKIN DISEASES.

THE main front of this building faces Quay Street, and here is the official entrance giving access to the board-room, house-surgeon's room, secretary's office, etc. In Byrom

GROUND FLOOR PLAN.

FIRST FLOOR PLAN.

SECOND FLOOR PLAN

Street are large gates opening into a forecourt, and in this court is the patients' entrance. On the ground floor of this part are the porter's room, telephone room, clerk's office, hall, and the "light" patients' waiting-room. In rear of the hall, and exactly opposite the vestibule, is a pentagonal bay which contains the main staircase, lavatories, etc. On the left of the staircase is the administration corridor leading to the Quay Street offices, and near this corridor is a second staircase in which the lift is incorporated. On the right is the corridor leading to the patients' waiting-room, consulting-room, dressing-rooms, bath-rooms, dispensary and lobby for the handing over of medicines. This out-patients' department may be looked upon as the chief part of the hospital, and its arrangements are all good. The waiting-room is of large size, and it is said that 200 patients can be accommodated in it.

On the first floor and over the out-patients' department are the Finsen Light room, the *x*-ray room, and the electric apparatus rooms. There is also a good well-ventilated hall for the "light" patients to wait in. The nurses' sitting-room occupies the space over the vestibule and porter's room. Over the administration part of the ground floor we have the nurses' dining-room, matron's rooms, linen-room, bath-rooms, and bedrooms for six nurses.

The second floor contains three wards for eight beds each, four single-bedded wards, two ward-kitchens, day-room, and bath-rooms and lavatories. There is also a room for sick nurses. The operating-room has attached to it a laboratory and an anæsthetic-room. Two of the eight-bedded wards have balconies and fire-escape staircases, and all the large wards have three windows on each side, and those facing Byrom Street have end windows as well. The closets and sinks attached to the wards are in all cases cut off from the main building by cross-ventilated passages.

That venerable bore "ancient lights" necessitated some modifications in the plans; but we give them as originally conceived by the architects, and, if we except the single-bedded wards from our judgment, the verdict on the arrangements generally must be, "very good, compact, and well thought out."

The architects are Messrs. Worthington and Sons, of Manchester.

## Figures and Tables

**Figure f1:**